# Dynamic alteration in miRNA and mRNA expression profiles at different stages of chronic arsenic exposure-induced carcinogenesis in a human cell culture model of skin cancer

**DOI:** 10.1007/s00204-021-03084-2

**Published:** 2021-05-25

**Authors:** Mayukh Banerjee, Ana Ferragut Cardoso, Laila Al-Eryani, Jianmin Pan, Theodore S. Kalbfleisch, Sudhir Srivastava, Shesh N. Rai, J. Christopher States

**Affiliations:** 1 Department of Pharmacology and Toxicology, University of Louisville, Louisville, KY USA; 2 Biostatistics and Bioinformatics Facility, James Graham Brown Cancer Center, University of Louisville, Louisville, KY USA; 3 Department of Biochemistry and Molecular Genetics, University of Louisville, Louisville, KY USA; 4 Department of Bioinformatics and Biostatistics, University of Louisville, Louisville, KY USA; 5 Centre for Agricultural Bioinformatics, ICAR-Indian Agricultural Statistics Research Institute, New Delhi, 110012 India; 6 Biostatistics and Informatics Facility, Center for Integrative Environmental Health Sciences, University of Louisville, Louisville, KY USA; 7 Present Address: Knowledge Management and Special Projects Branch, Center for Strategic Scientific Initiatives (HNC1L), National Cancer Institute, National Institutes of Health, Bethesda, MD 20892 USA; 8 Present Address: Gluck Equine Research Center, University of Kentucky, Lexington, KY USA

**Keywords:** Arsenic, Skin carcinogenesis, Differential gene expression, Pathway analysis, Passage matching, Endoplasmic reticulum stress

## Abstract

Chronic arsenic exposure causes skin cancer, although the underlying molecular mechanisms are not well defined. Altered microRNA and mRNA expression likely play a pivotal role in carcinogenesis. Changes in genome-wide differential expression of miRNA and mRNA at 3 strategic time points upon chronic sodium arsenite (As^3+^) exposure were investigated in a well-validated HaCaT cell line model of arsenic-induced cutaneous squamous cell carcinoma (cSCC). Quadruplicate independent HaCaT cell cultures were exposed to 0 or 100 nM As^3+^ for up to 28-weeks (wk). Cell growth was monitored throughout the course of exposure and epithelial-mesenchymal transition (EMT) was examined employing immunoblot. Differentially expressed miRNA and mRNA profiles were generated at 7, 19, and 28-wk by RNA-seq, followed by identification of differentially expressed mRNA targets of differentially expressed miRNAs through expression pairing at each time point. Pathway analyses were performed for total differentially expressed mRNAs and for the miRNA targeted mRNAs at each time point. RNA-seq predictions were validated by immunoblot of selected target proteins. While the As^3+^-exposed cells grew slower initially, growth was equal to that of unexposed cells by 19-wk (transformation initiation), and exposed cells subsequently grew faster than passage-matched unexposed cells. As^3+^-exposed cells had undergone EMT at 28-wk. Pathway analyses demonstrate dysregulation of carcinogenesis-related pathways and networks in a complex coordinated manner at each time point. Immunoblot data largely corroborate RNA-seq predictions in the endoplasmic reticulum stress (ER stress) pathway. This study provides a detailed molecular picture of changes occurring during the arsenic-induced transformation of human keratinocytes.

**Supplementary Information:**

The online version contains supplementary material available at 10.1007/s00204-021-03084-2.

## Introduction

Chronic exposure to arsenic, a naturally occurring metalloid, adversely affects over 225 million people worldwide (Naujokas et al. 2013; Podgorski and Berg 2020). Arsenic is a Class I multi-organ carcinogen in humans (IARC. 2012), skin being the most common target organ (Karagas et al. 2015). Arsenic exposure is the second leading cause of skin cancer after UV in sunlight (Surdu 2014). Arsenic-induced cutaneous squamous cell carcinoma (cSCC) is common in exposed human populations (Banerjee 2011) and is extremely invasive with a high rate of recurrence and fatality (Waldman and Schmults 2019). Although studied extensively, molecular mechanisms of arsenic-induced skin carcinogenesis are still controversial [reviewed in (Hunt et al. 2014)].

Lack of animal model systems has hindered understanding the events leading up to chronic arsenic exposure induced skin cancers. It has never been possible to induce skin cancer in rodents with arsenic exposure alone, even at exposures of 50 ppm (Tokar 2016), which far exceeds the environmental exposure levels human populations encounter (Ghosh et al. 2007; Gonsebatt et al. 1994; Nigra et al. 2020).

Epidemiological studies demonstrate that mean blood arsenic levels of human populations chronically exposed to 100–300 ppb arsenic in drinking water is about 100 nM (Gonsebatt et al. 1994, 1997; Pi et al. 2000). Primary human skin keratinocyte lines transformed through chronic exposure to in vivo levels induced by environmentally relevant levels of arsenite (As^3+^) would be ideal to study the molecular mechanisms involved in arsenic-induced cSCC. Unfortunately, primary cell lines undergo only 16–20 passages before they become senescent, too short for chronic exposure regimes. The other option is to study skin tissues from exposed human populations. A handful of studies, including from our group, have employed this approach to dissect the underlying mechanisms of arsenic-induced skin cancers (Al-Eryani et al. 2018a; Guo et al. 2016). However, one inherent issue with such studies is, they look at the endpoint after the transformation has occurred, leaving a substantial knowledge gap in understanding the molecular events leading up to a transformed phenotype. In absence of this knowledge, it is difficult to understand if the changes observed are causes or effects of transformation.

Most of the current knowledge about arsenic carcinogenesis is gained from studies on cell line models treated with arsenic. Unfortunately, many of the studies have used high arsenic concentrations (micromolar to millimolar) and short exposure duration (hours to days). Arsenic is well known to have hormetic effects upon a variety of molecules and cellular pathways (Calabrese and Baldwin 2003; Hashmi et al. 2014) making it difficult to extrapolate the results meaningfully to chronically exposed populations. Moreover, most of these studies look at one or few endpoints at the end of the exposure regime. Thus, they offer only a limited view of the molecules and pathways that represent a small fraction of complex interactions and dysregulation of multiple networks occurring simultaneously upon As^3+^ exposure.

Fortunately, immortalized human keratinocytes (HaCaT) are an in vitro model for normal human keratinocytes (Boukamp et al. 1988) that can be used to study arsenic-induced skin cancer (Pi et al. 2008). Pi et al*.* showed that continuous exposure of HaCaT cells for 28-wk to a low level (100 nM) of sodium arsenite transformed these cells and resulted in an aggressive squamous cell carcinoma phenotype upon inoculation of nude mice (Pi et al. 2008). Previous studies in our laboratory showed that chronic As^3+^ exposure for 7-wk led to differential small RNA and mRNA expression (Al-Eryani et al. 2017). HaCaT cells chronically exposed to 100 nM NaAsO_2_ were observed to grow slower until 19–20-wk when transformation starts (Pi et al. 2008; Sun et al. 2009). A third important time point to study in the transformation path is 28-wk by which time cells are transformed (Pi et al. 2008). Importantly, the As^3+^ exposure used reflects the actual mean blood arsenic levels of chronically exposed populations (Gonsebatt et al. 1994, 1997; Pi et al. 2000), and is thus toxicologically and environmentally relevant. This well-established model thus provides an excellent opportunity to explore the molecular events happening as a function of time starting from the initiation of exposure through to the cells acquiring cSCC phenotype. The current study aimed to provide an understanding of the complex interactions of miRNA, mRNA and the pathways and networks they regulate at these three critical times during chronic As^3+^ exposure-induced cSCC.

Thus, a longitudinal study was performed to determine miRNA and mRNA differential expression in HaCaT cells chronically exposed to 100 nM NaAsO_2_ at 3 time points: 7-wk (early transformation related changes), 19-wk (start of actual transformation) and 28-wk (fully transformed cells). Employing a state-of-the-art RNA-seq platform, we show that at each time point, a considerable number of miRNAs and mRNAs are being differentially regulated in As^3+^-exposed HaCaT cells compared to passage-matched unexposed cells. Through expression pairing analysis, we show that the differentially expressed mRNAs at each time point are targeted by the differentially expressed miRNAs at that time point. Furthermore, we demonstrate that at each time point pathways known to be involved in carcinogenesis are being dysregulated and that these dysregulated pathways interact extensively as complex networks. Furthermore, we validate the RNA-seq predictions at the protein level through immunoblot for several molecules involved in the endoplasmic reticulum (ER) stress pathway. Our study reflects the dynamic and temporally labile mechanisms by which major biochemical pathways are regulated by chronic As^3+^ exposure ultimately leading to cSCC.

## Materials and methods

### Chemicals

Sodium arsenite (NaAsO_2_; CAS 7784–0698) was obtained from Thermo Fisher Scientific Inc. (Waltham, MA, USA). Single-thaw aliquots of sodium arsenite were prepared in UltraPure™ DNase/RNase-Free Distilled Water (Thermo Fisher Scientific Inc.) and were thawed immediately before use. MEM alpha modification media, trypsin, ethylene diamine tetraacetic acid and penicillin/ streptomycin were obtained from Thermo Fisher Scientific Inc. Fetal Bovine Serum (characterized) was obtained from Hyclone (Logan, UT, USA). All other chemicals were obtained from Thermo Fisher Scientific Inc., unless specifically mentioned.

### Cell Culture

The HaCaT model of Pi et al*.* (2008) was adopted for the present study. HaCaT cells were the kind gift of Dr. TaiHao Quan, University of Michigan. HaCaT cell cultures were maintained as independent quadruplicates (4 with and 4 without 100 nM NaAsO_2_) for 28-wk. The cells were cultured in MEM alpha modification media supplemented with 10% fetal bovine serum, 100 units/mL penicillin/100 µg/mL streptomycin and 2 mM glutamine. Cultures were maintained at 37 °C in a humidified 5% CO_2_ atmosphere. Cells were passaged twice a week and 10^6^ cells were plated per 100 mm dish every time. At each passage, the total cells were counted for calculation of population doubling. Identity of cultures as HaCaT cells was confirmed by STR mapping (Genetica, Burlington, NC).

### Total RNA isolation

In order to isolate total RNA (including mRNA and small RNA) from cells harvested at 7, 19 and 28-wk time points, mirVana™ RNA isolation kit was employed as described previously (Al-Eryani et al. 2018b) following the manufacturer’s recommendations. The quality of the isolated RNA was determined using the Agilent RNA 6000 Pico Kit, Eukaryote, version 2.6 and the Agilent 2100 Bioanalyzer instrument (Agilent Technologies, Inc., Santa Clara, CA, USA). All samples used had RIN (RNA integrity number) > 9.

### miRNA library preparation, cluster generation and sequencing

Library preparation, cluster generation and sequencing of all 24 samples were performed in the Center for Genetics and Molecular Medicine (CGeMM) DNA Facility Core at the University of Louisville. The Truseq Small RNA kit was used to prepare miRNA libraries from 1 μg total RNA. Each Library was individually gel purified on a Novex TBE 6% gel and resuspended in 10 μL 10 mM Tris–HCl, pH 8.5. Libraries were subsequently validated and quantitated by running 1 μL sample on the Agilent Technologies 2100 Bioanalyzer DNA High Sensitivity Chip. Thirty-six-cycle single sequencing reads were generated on the Illumina NextSeq500 instrument utilizing the 500 High output v2 (75 cycle) sequencing kit.

### mRNA library preparation, cluster generation and sequencing

The Truseq Stranded mRNA kit was used to prepare mRNA libraries from 1 μg total RNA. Libraries were validated on the Agilent 2100 Bioanalyzer and quantitated using the Illumina Library Quantification Kit, ABI Prism qPCR Mix from Kapa Biosystems and the ABI7900HT real-time PCR instrument. All samples were pooled and run simultaneously on 4 flow cells, using 2 × 150 paired end sequencing with the 500 High-output v2 (300 cycles) sequencing kit on the Illumina NextSeq500 instrument.

### Data mapping

Paired end RNA-Seq data were generated for each experimental condition. The data for each replicate were stored, trimmed, mapped, and quantified individually. For miRNA, the newly generated reads were trimmed using Trimgalore (Martin 2011) followed by pre-processing and subsequent analysis using miRDeep2 v:0.0.8 (Friedlander et al. 2012). The trimmed reads were mapped to the human reference genome hg19 (NCBI build 37.1 released 2009) using the RNA-Seq mapping software TopHat (Trapnell et al. 2012) and annotated transcripts were quantified in units of Fragments Per Kilobase of transcript per Million mapped reads (FPKM) using Cufflinks (Trapnell et al. 2012). The gene annotation used for the quantification was downloaded from ENSEMBL (version 81). All the ribosomal RNA (rRNA) and mitochondrial tRNA were removed to ensure that they would not influence the derived FPKM values. Further details on the pipeline and algorithms used for data mapping and analysis are provided in the Supplementary Methods. Data have been deposited in the GEO database, accession number GSE153057. For analysis of differential expression of mRNA, the RNA seq data was first checked for quality control using FastQC v0.11.8 (Wingett and Andrews 2018). Data trimming was done using Trimmomatic version 0.38 (Bolger et al. 2014). Sequence alignment was performed by Star 2.6 (Dobin et al. 2013; Dobin and Gingeras 2015) against human genome reference from Ensembl (Release 95) (Schneider et al. 2017; Zerbino et al. 2018). Feature count was done using the R package “Rsubread” (Liao et al. 2013). Data have been deposited in the GEO database, accession number GSE107054.

### RNA-Seq data analysis

For miRNA, data analysis was performed by comparing the log values of the counts generated from the sample reads + 0.00001 of exposed cells to the passage matched unexposed cells at each time point tested. The fold changes of the compared values were calculated using the equation: FC = mean (Exposed counts + 0.00001)/mean (Unexposed counts + 0.00001). The *p*-values for these comparisons were calculated by two sample t-Test with equal variances (p_Eq). Differentially expressed miRNA at each time point was defined as p_Eq < 0.05. All analyses were obtained using SAS System V9. Cary, NC: SAS Institute Inc, 2003. For differential expression of mRNA, first genes having 0 values in all the samples for a single time point were removed from the dataset. The remaining data were analyzed for differential gene expression employing the R package "edgeR" (McCarthy et al. 2012; Robinson et al. 2010). Trimmed mean of M values (TMM) was used for normalization of the data for all the comparisons (Robinson and Oshlack 2010), while exact test (exactTest function) was employed for testing the significance of the difference in gene expression (Robinson and Smyth 2008). Differentially expressed mRNA molecules were defined as *p* < 0.01 and FC >  ± 30% compared to passage-matched unexposed HaCaT cells.

### Pathway analyses

Differentially expressed miRNA (p_Eq < 0.05) and mRNA molecules (*p* < 0.01 and FC >  ± 30%) were analyzed by Ingenuity® Pathway Analysis (IPA®) (Qiagen Inc.). IPA® core analysis was performed on the differentially expressed mRNA dataset to generate a prediction of dysregulated canonical pathways at each time point. Pathways with − log(p-value) > 1.3 and IZ-scoreI >  ± 1 was used to predict activation/inhibition status of the pathways. Z-score > 1 was activated, while Z-score < -1 was inhibited. Furthermore, differentially expressed mature miRNA molecules at each time point (as identified by IPA®) were expression paired with differentially expressed mRNA molecules at each time point to identify differentially expressed mRNA targets of differentially expressed miRNA molecules at each time point. IPA® core analysis was subsequently performed on the differentially expressed mRNA targets of differentially expressed miRNAs dataset to generate a prediction of dysregulated canonical pathways at each time point. Figures for pathway interactions based on our differentially expressed mRNA data were prepared using IPA®^.^

### Immunoblotting

Immunoblotting was performed to examine EMT and the protein expression status of selected ER stress pathway molecules at all three-time points. Sample preparation, estimation of protein content, and immunoblotting, and image acquisition was performed as described previously (Banerjee et al. 2020). Details regarding the antibodies used and their dilutions are presented in Supplementary Table 1. Raw data for densitometric analysis was generated from the images employing Image J software (Schneider et al. 2012).

### Statistical analysis

Cell population doublings were calculated from total cell numbers for each independent culture at each passage. Mean cumulative doubling number (along with SD as a measure of error) for passage matched unexposed and As^3+^ exposed samples were plotted against time to generate the growth curve. The data were analyzed using R (R-Core-Team 2018) by two-way ANOVA followed by Tukey's post-hoc test; *p*-value ≤ 0.05 was considered significant (Tukey 1949). Densitometric data for EMT and ER stress markers were analyzed using an unpaired two-tailed *t*-test; *p*-value ≤ 0.05 was considered significant. For each molecule at each time point, the mean of the unexposed samples was set to 100% and data were expressed as % mean unexposed at that time point. Densitometric analysis, heat maps, and bar graphs were generated using GraphPad Prism 9.0.1 (GraphPad Software, San Diego, CA, USA). Venn diagrams were generated employing the Venn Diagram Plotter program (https://omics.pnl.gov/software/venn-diagram-plotter).

## Results

### As^3+^ exposure alters the growth curve pattern and induces EMT

**Fig. 1:**
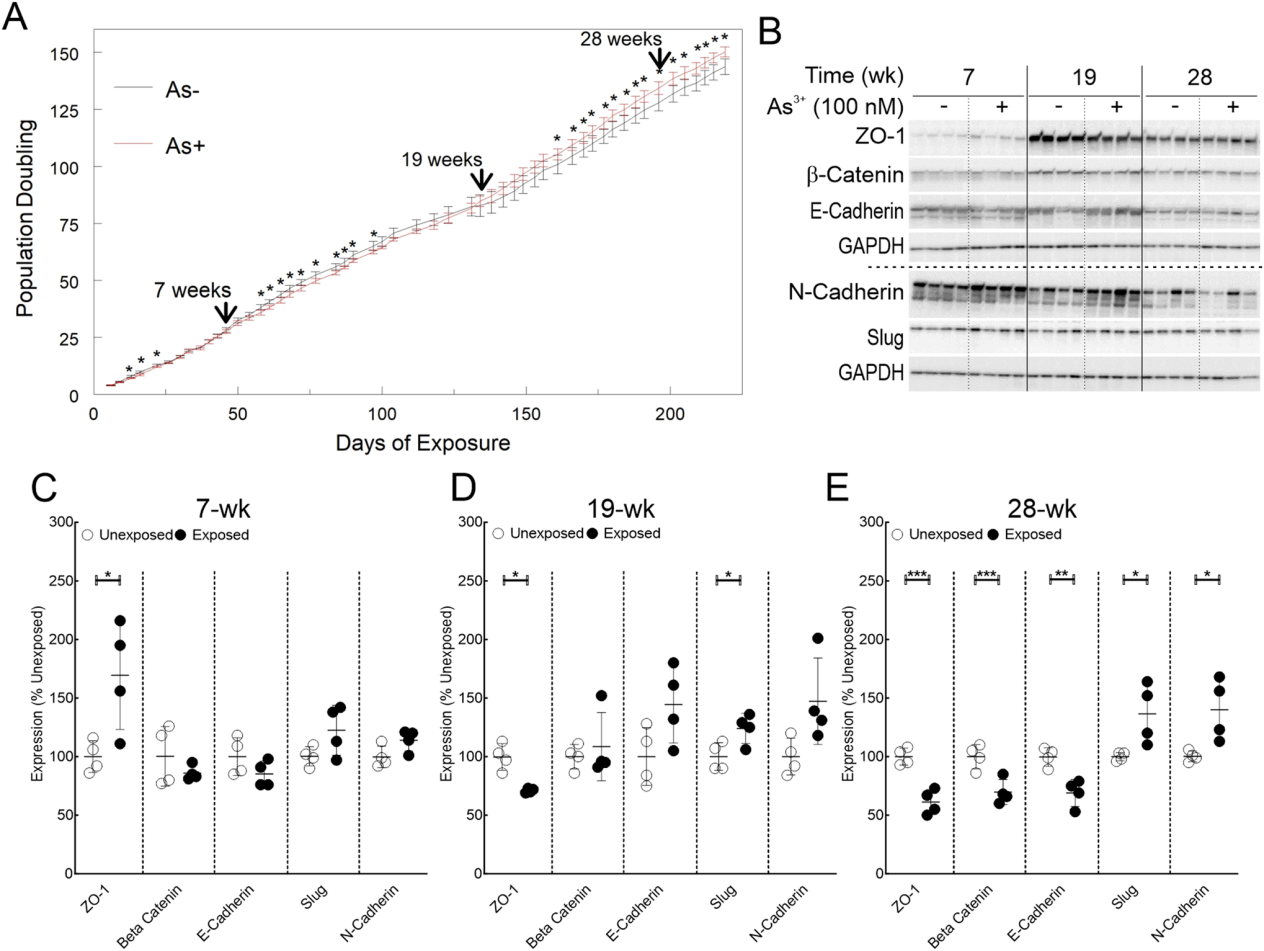
Chronic As^3+^ exposure leads to altered growth rate and EMT. **A** Impact of As^3+^ exposure on cumulative HaCaT cell population doubling. Quadruplicate independent HaCaT cell cultures were incubated with 0 or 100 nM As^3+^_._ Population doublings were calculated and the means ± SD of cumulative doubling at each passage were plotted. Statistical analysis was done by two-way ANOVA, *p ≤ 0.05. **B** Immunoblot for EMT markers at 7, 19 and 28-wk time points in HaCaT cells exposed to 100 nM As^3+^ or passage matched unexposed controls. **C** Densitometric analysis of EMT marker expression at 7-wk. **D** Densitometric analysis of EMT marker expression at 19- wk. **E** Densitometric analysis of EMT marker expression at 28-wk. Protein expression data in panels **C**-**E** are plotted as means ± SD and expressed as % mean unexposed. Statistical analysis was done by unpaired two-tailed *t*-test; *p ≤ 0.05, **p ≤ 0.01; ***p ≤ 0.001

The growth curve data are represented in Fig. 1A. Comparison of mean cumulative cell doubling in As^3+^-exposed HaCaT cells to corresponding passage matched unexposed controls revealed that growth rates of As^3+^ exposed cells were slower at several times points prior to 19-wk. At 19-wk, the growth rates of As^3+^ exposed cells became similar to passage matched unexposed cells, while at later time points, As^3+^-exposed cells grew significantly faster. In conjunction with changes in the growth pattern, As^3+^-exposed cells had undergone EMT upon 28-wk of exposure (Fig. 1B–E) as demonstrated by suppression of epithelial markers (ZO-1, β-Catenin and E-Cadherin) and concomitant induction of mesenchymal markers (Slug and N-Cadherin). Interestingly, even at 19-wk of exposure, ZO-1 was suppressed, and Slug induced (Fig. 1B, D). These data together with alteration in cell growth rate strengthen the notion that As^3+^-exposed cells begin transforming around 19-wk.

### As^3+^ exposure induces differential miRNA and mRNA expression pattern with time

**Fig. 2:**
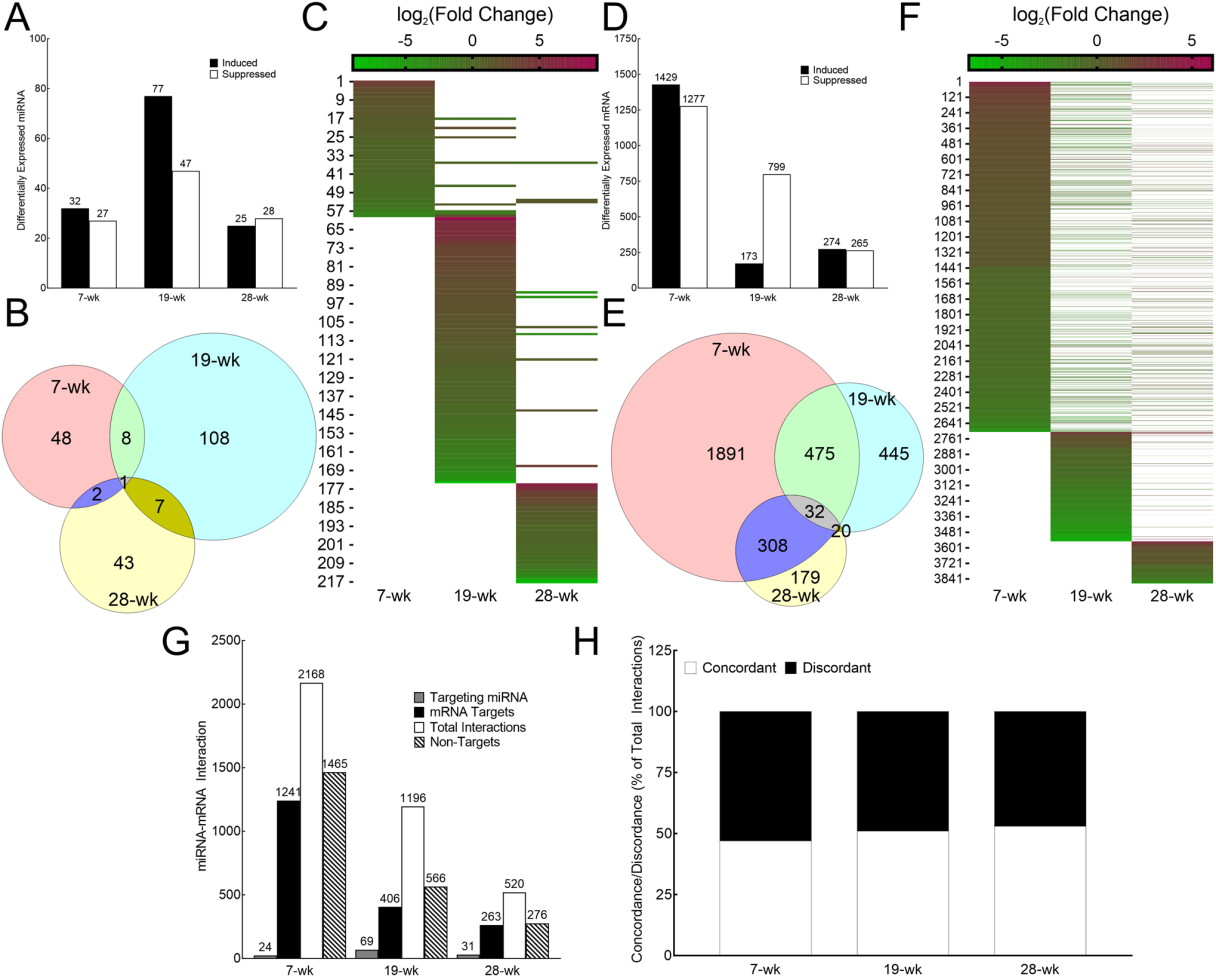
Chronic As^3+^ exposure changes the landscape of differentially expressed miRNAs and mRNAs in a longitudinal manner. **A** Bar graph showing the number of miRNA molecules at 7, 19 and 28-wk time points differentially expressed in HaCaT cells exposed to 100 nM As^3+^ compared with passage matched unexposed controls (induced: closed bars; suppressed: open bars). Differential expression is defined as p_Eq < 0.05. **B** Venn diagram depicting the distribution of differentially expressed miRNAs (p_Eq < 0.05) at each time point along with the number of overlaps at different time points. **C** Heat Map of differentially expressed miRNA molecules at 7, 19 and 28-wk. The numerals on the y axis refers to the serial numbers assigned to the differentially expressed miRNA molecules in Supplementary Table 2. The color code bar on top refers to the log_2_ (Fold Change) expression values. Absence of a bar (represented by white) signifies either that miRNA molecule was not detected at that time point or was not differentially expressed at that time point. **D** Bar graph showing the number of mRNA molecules at 7, 19 and 28-wk time points differentially expressed in HaCaT cells exposed to 100 nM As^3+^ compared with passage matched unexposed controls (induced: closed bars; suppressed: open bars). Differential expression is defined as p < 0.01 and FC ±  > 30%. **E** Venn diagram depicting the distribution of differentially expressed mRNAs (*p* < 0.01 and FC ±  > 30%) at each time point along with the number of overlaps at different time points. **F** Heat Map of differentially expressed mRNA molecules at 7, 19 and 28-wk. The numerals on the y axis refers to the serial numbers assigned to the differentially expressed miRNA molecules in Supplementary Table 3. The color code bar on top refers to the log_2_ (Fold Change) expression values. Absence of a bar (represented by white) signifies either that mRNA molecule was not detected at that time point or was not differentially expressed at that time point. **G** Expression pairing between differentially expressed miRNA and differentially expressed mRNA at 7, 19 and 28-wk. For each time point, shown are the total number of differentially expressed miRNA molecules that are targeting one or more differentially expressed mRNA molecules (light grey bars); the number of differentially mRNA molecules that are targeted by one or more or differentially expressed miRNA molecules (closed bars), total number of miRNA-mRNA pairings (open bars) and the number of differentially expressed mRNA molecules that are not predicted to be targeted by any differentially expressed miRNA molecule (hatched bars). **h** Concordance–discordance relationship between differentially expressed miRNA and differentially expressed mRNA at 7, 19 and 28-wk. The data is presented as % of total interactions predicted at that time point

More than 50 miRNAs were differentially expressed between As^3+^-exposed and unexposed HaCaT cells at each time point, with the most (124) at 19-wk (Fig. 2A–C). Data on individual differentially expressed miRNAs at each time point are presented in Supplementary Table 2. Longitudinal comparison showed that most of the differentially expressed miRNAs were unique to one-time point (Fig. 2B–C) only, with relatively few miRNAs being differentially expressed at two-time points (irrespective of induction or suppression status). Only one miRNA (hsa-miR-6733) was represented at all three-time points and was consistently suppressed (Fig. 2B–C; Supplementary Table 2). These data indicate that the miRNA landscape is markedly different at each phase of transformation.

Examination of the mRNA data revealed that 2706, 972 and 539 molecules were differentially expressed in As^3+^ exposed cells compared to passage matched unexposed cells at 7, 19 and 28-wk time points respectively (Fig. 2D–F). Data on individual differentially expressed mRNAs at each time point is presented in Supplementary Table 3. The longitudinal comparison showed that most of the differentially expressed miRNAs were unique to 7 and 19-wk time points, while those differentially expressed at 28-wk time point had considerable overlap with those at 7-wk time point but not with 19-wk time point (Fig. 2E–F). As seen for miRNAs, the mRNA data indicate that the mRNA landscape is markedly different at each phase of transformation.

We were further interested in examining if the differentially expressed miRNA molecules could explain the differentially expressed mRNA molecules at each time point. Expression pairing analysis demonstrated that 42–49% of the differentially expressed mRNA molecules are being targeted by the differentially expressed miRNAs depending on the time point (Fig. 2G). Several differentially expressed mRNA molecules at each time point can be targeted by more than one differentially expressed miRNA, resulting in 2168, 1196, and 520 miRNA-mRNA pairings at 7, 19, and 28-wk time points respectively (Fig. 2G). Individual miRNA-mRNA pairing data at each time point is provided in Supplementary Table 4. As shown in Fig. 2H, at each time point ~ 50% of the pairings were found to be concordant (inverse expression relationship between differentially expressed miRNA and its differentially expressed target mRNA). It was also found that several differentially expressed mRNA molecules at each time point were being targeted by several differentially expressed miRNA molecules (Supplementary Table 4), which, in part could explain some of the discordance in the dataset.

### As^3+^ exposure dysregulates multiple canonical pathways in a temporally dynamic manner

**Fig. 3:**
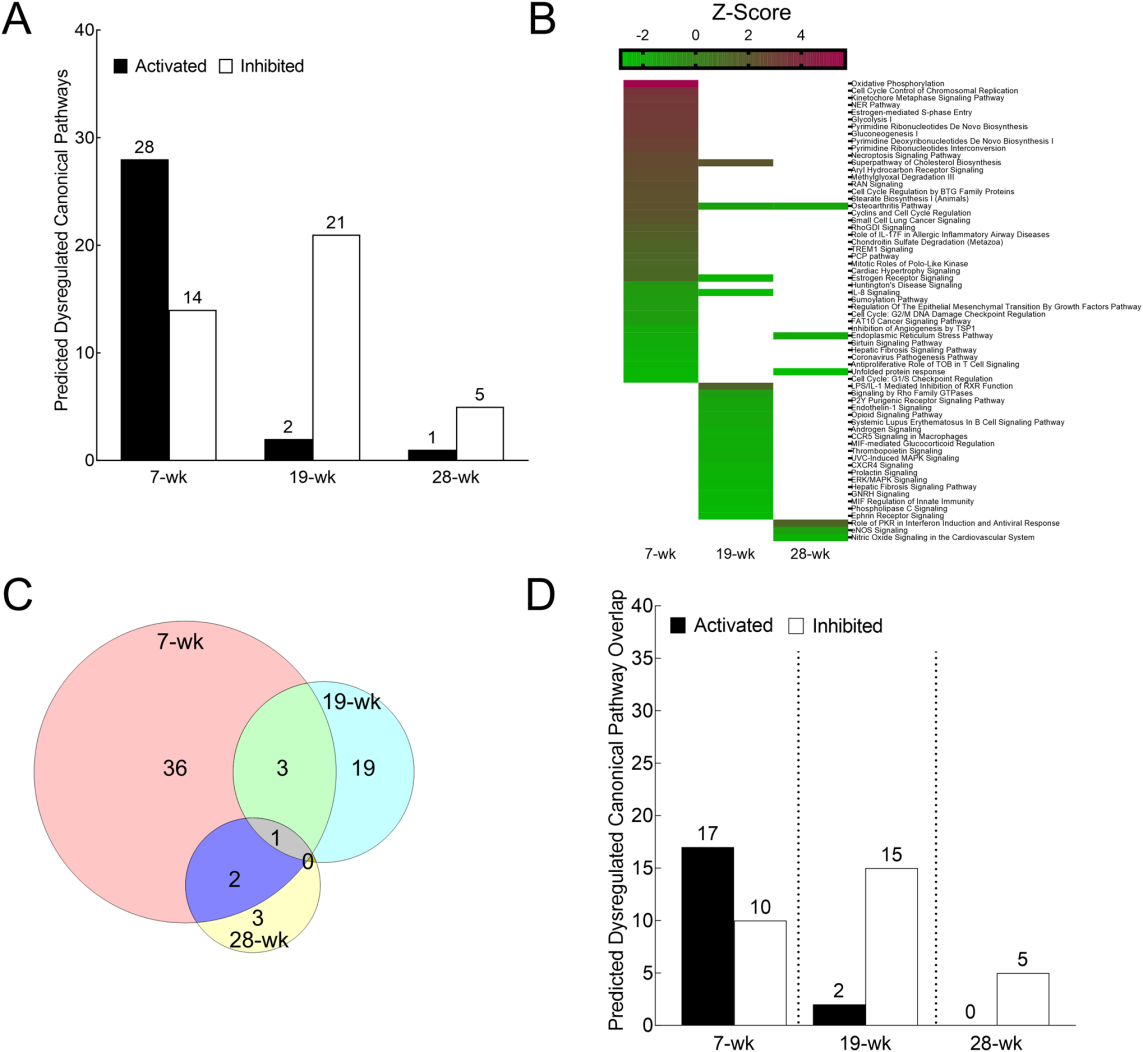
Chronic As^3+^exposure leads to widespread dysregulation in predicted canonical pathways in a longitudinal manner. **A** Bar graph showing the number of predicted dysregulated pathways at 7, 19 and 28-wk time points in HaCaT cells exposed to 100 nM As^3+^ compared with passage matched unexposed controls (activated: closed bars; inhibited: open bars). Activation is defined as -log(p-value) > 1.3; Z-score > 1, while inhibition is defined as -log(p-value) > 1.3; Z-score < -1. **B** Heat Map of predicted activated/inhibited pathways based on all differentially expressed mRNA molecules at 7, 19 and 28-wk (presented in the same order as in Supplementary Table 5). The color code bar on top refers to the Z-score values. Absence of a bar (represented by white) signifies that the pathway was not predicted to be activated or inhibited at that time point. **C** Venn diagram depicting the distribution of predicted dysregulated pathways [-log(*p*-value) > 1.3; Z-score >  ± 1] at each time point along with the number of overlaps at different time points. **D** Bar graph showing the number of pathways at 7, 19 and 28-wk time points predicted to be activated/inhibited both by the differentially expressed mRNA dataset (presented in Supplementary Table 5) as well as the differentially expressed mRNA targets of differentially expressed miRNA dataset (Presented in Supplementary Table 6)

IPA® analysis of differentially expressed mRNAs revealed numerous biochemical pathways to be activated/inhibited upon chronic As^3+^ exposure at each time point (Fig. 3A). The activation/inhibition status of the predicted dysregulated pathways based on the differentially expressed mRNA molecules at each time point are depicted in Fig. 3B and Supplementary Table 5. The longitudinal comparison demonstrates that most of the predicted dysregulated pathways at 7 and 19-wk time points are not dysregulated at other time points. However, 2 of the 6 predicted dysregulated pathways at 28-wk are also dysregulated at 7-wk, while 1 more (osteoarthritis pathway) is dysregulated at all three timepoints (Fig. 3B–C) and is activated at 7 wk but inhibited at the two later time points. These data are consistent with the mRNA data indicating that the gene expression landscape is markedly different at each time during transformation.

Next, we wanted to explore if differentially expressed mRNA targets of differentially expressed miRNA molecules would be consistent with these dysregulated pathways predictions of total differentially expressed mRNAs. Therefore, we performed a nested pathway analysis based only on the differentially expressed mRNA that were targeted by differentially expressed miRNA at each time point (Supplementary Fig. 1A–B). Pathways predicted to be activated/inhibited based on the subset of differentially expressed mRNA targeted by differentially expressed miRNA (Supplementary Table 6) demonstrated 65–81% overlap with the pathways predicted based on entire differentially expressed mRNA data depending on the time point (Fig. 3D). More detailed comparison of the pathways predicted based on the two analyses is presented in Supplementary Fig. 1C.

### Corroboration of RNA-Seq and pathway analysis prediction of ER stress pathway inhibition at the protein level

ER stress pathway and its interaction with unfolded protein response are well characterized to play pivotal role in carcinogenesis (Chen and Cubillos-Ruiz 2020). Therefore, the ER stress pathway was selected to validate the RNA-seq data and pathway analysis predictions at the protein level. This pathway is predicted to be inhibited at both 7 and 28-wk, but not at 19-wk in both the total mRNA dataset analysis as well as the subset analysis (Fig. 3B, C; Supplementary Tables 5 and 6). Furthermore, while the pathway was predicted to be inhibited at two-time points, several molecules were differently modulated between the two-time points (Supplementary Fig. 2). This provided an ideal opportunity to examine if the RNA-seq data and the IPA pathway predictions can be validated at the level of protein expression. To assess if the RNA-seq predictions hold true at the protein level, we selected 8 molecules in the ER stress pathway as shown in Supplementary Fig. 2 (ATF4, BCL2, BIP, CHOP, IRE1, HSP72, NRF2 and PERK).

**Fig. 4:**
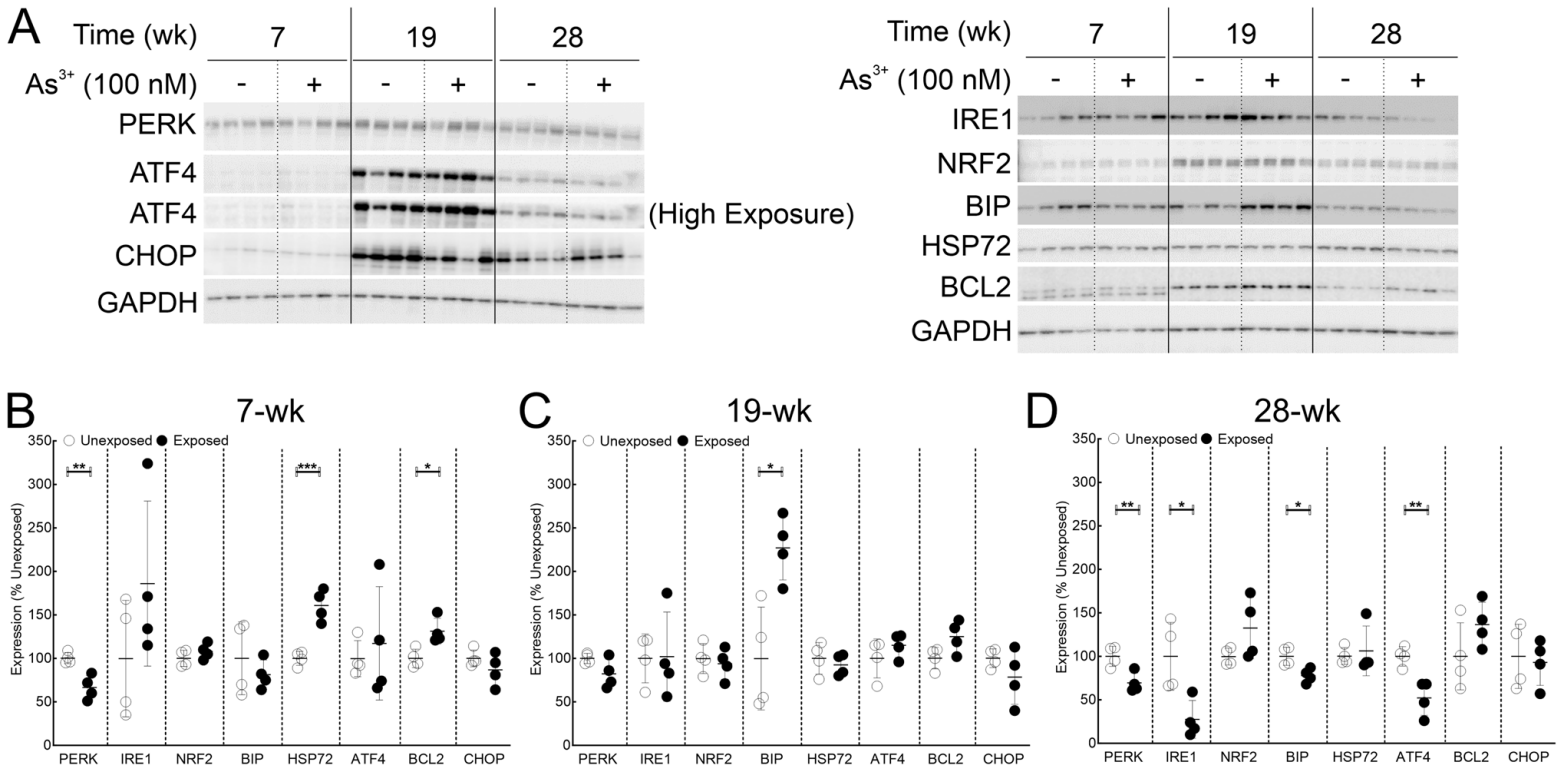
Immunoblot analysis validates dysregulation of ER stress pathway predicted by RNA-seq data analyses at the protein level. **A** Immunoblot for ER stress markers at 7, 19 and 28-wk time points in HaCaT cells exposed to 100 nM As^3+^ or passage matched unexposed controls. **B** Densitometric analysis of ER stress marker expression at 7-wk. **C** Densitometric analysis of ER stress marker expression at 19-wk. **D** Densitometric analysis of ER stress marker expression at 28-wk. Protein expression data in panels **B**–**D** are plotted as means ± SD and expressed as % mean unexposed. Statistical analysis was done by unpaired two-tailed *t*-test; *p ≤ 0.05, **p ≤ 0.01; ***p ≤ 0.001

Immunoblot data for ER stress markers at three time points presented in Fig. 4A. Several of the proteins analyzed show time-dependent expression patterns in addition to As^3+^-dependent expression highlighting the importance of passage-matched controls. Densitometric analysis (Fig. 4B-D) corroborates the RNA seq predictions for 4 out of 8 molecules at all three-time points (ATF4, HSP72, NRF2 and PERK), 2 molecules at two-time points (BCL2 at 7 & 28-wk and CHOP at 7 & 19-wk) and remaining 2 molecules for one-time point each (BIP at 28-wk and IRE1 at 19-wk). These results support the predictions made by analyses of the RNA data.

## Discussion

Mechanisms of chronic arsenic-exposure-induced carcinogenesis are widely studied, but a clear picture is far from emerging. A multitude of mechanisms has been implicated and debated for their putative role in arsenic-induced carcinogenesis (Chen and Costa 2018; Cohen et al. 2016; Huang et al. 2019; Lee and Yu 2016; States 2015; Zhou and Xi 2018). It is well established that transcriptomic and proteomic profiles are globally altered in arsenic-induced cancer tissues or in arsenic-transformed cell lines (Guo et al. 2016; Mir et al. 2017). Furthermore, experimental data from our group and others indicate that such genome-wide differential expression might be regulated partly by differential expression of miRNAs (Al-Eryani 2017; Al-Eryani et al. 2018a; Bustaffa et al. 2014; Cardoso et al. 2018). However, a considerable knowledge gap exists in understanding the molecular processes that are operative in the interim between the beginning of exposure and the time cells are fully transformed.

The current study aims to fill this gap in the existing literature by providing a comprehensive picture of the molecular events at three stages of arsenic-induced carcinogenesis (early transformation related changes at 7-wk, transformation initiation at 19-wk and fully transformed at 28-wk). For this purpose, we combined longitudinal study design using passage-matched independent quadruplicate biological replicates along with large-scale genome-wide RNA-seq platform. This approach ensured that the analyses have considerable power to rule out random stochastic events as false positives or negatives while generating high-quality in-depth data. The passage matching was an important aspect in our study to rule out the effect of long passaging time. It is evident that several molecules follow a temporal variation in expression pattern irrespective of the exposure status (ATF4, BCL2, IRE1, NRF2 and CHOP in Fig. 4). While the reasons for this observation are not clear, it is certain that without appropriate passage matching, the robustness of our analyses would have been seriously compromised.

We unequivocally demonstrate temporally modulated changes in the growth patterns in the As^3+^ exposed HaCaT cells (Fig. 1) in agreement with data from other groups (Pi et al. 2008; Sun et al. 2009). The slower growth rates in As^3+^ exposed cells at early stages of exposure could reflect the well-characterized cell cycle arrest/delay effects of As^3+^ exposure (Al-Eryani et al. 2017; States 2015; States et al. 2002; Tam et al. 2020). This inference is supported by the pathway analysis which shows a multitude of cell cycle related pathways being dysregulated at 7-wk (Fig. 3B, Supplementary Table 5 and Supplementary Fig. 2). In the next phase between 7 and 19-wk, there is a clear switch in the growth rate and a concomitant reversal of expression pattern of epithelial marker ZO-1 and mesenchymal marker Slug (Fig. 1B, D) which signals the initiation of EMT. Finally, at 28-wk, the growth rate of As^3+^-exposed cells far exceeds that of passage matched unexposed controls, along with the reversal of the expression profiles of all the EMT markers tested (Fig. 1B, E), telltale signatures of carcinogenic transformation (Brabletz et al. 2018; Mehrara et al. 2007).

Our RNA-seq analyses show that multitudes of mRNAs and miRNAs targeting those mRNAs are differentially expressed in a time dependent manner (Fig. 2). Interestingly, the majority of the differentially expressed miRNA and mRNA molecules at each time point (corresponding to different stages in cancer development) are largely unique, as are the predicted activated/inhibited pathways they populate (Fig. 2 and Fig. 3). This limited longitudinal overlap between the differentially expressed miRNAs or mRNAs suggest that the changing landscape of differentially expressed miRNAs might be contributing considerably to the changing landscape of differentially expressed mRNAs at each time point. Moreover, the mRNA expression alone may be a poor surrogate to understand exactly the impact of these changes in mRNA expression. Secondly, pathway analyses of differentially expressed mRNA targets of differentially expressed miRNAs at each time point show a great degree of overlap in the predicted canonical pathways with those of all differentially expressed mRNAs at those time points (Fig. 3E). Consequently, it is reasonable to hypothesize that the predicted alterations in the canonical pathways are largely guided by differential expression of mRNAs that are being targeted by the differentially expressed miRNAs at that time point. Thirdly, the fact that analysis of a subset of the differentially expressed mRNAs produces results similar to that of the entire dataset points to the robustness of the data and the analyses performed. Taken together, it is possible that at each stage, a new set of key molecules are being induced/suppressed that could be bringing about molecular changes associated with the sequential process of carcinogenesis.

Interestingly, only one pathway (osteoarthritis pathway) was found to be dysregulated at all three-time points (activated at 7-wk, inhibited at 19 and 28-wk) both in the differentially expressed mRNA (Fig. 3B) as well as differentially expressed mRNA targets of differentially expressed miRNA datasets (Supplementary Fig. 1). Furthermore, one more pathway (hepatic fibrosis signaling pathway) was found to be inhibited across all three-time points in the differentially expressed mRNA targets of differentially expressed miRNA datasets (Supplementary Fig. 1). While these pathways apparently have little to do with skin carcinogenesis, a closer look at the dysregulated molecules yield some interesting insights (Supplementary Fig. 3). Several of the dysregulated molecules, including molecules in the MAP kinase pathway, Rho signaling, TGF-β signaling, and MMPs, are involved in multiple cellular processes and are well-known players in the carcinogenesis of multiple organs (Derynck et al. 2021; Gialeli et al. 2011; Goel and Mercurio 2013; Huebner et al. 2019; Vella et al. 2018).

However, the question remains how these changes might usher in the changes that ultimately culminate in carcinogenesis. Pathway analysis data from this study shed some interesting light in this regard. Several of the top predicted canonical pathways at each time point reflect well-established mechanisms previously implicated in arsenic-induced carcinogenesis. At 7-wk, there is clear evidence of dysregulation of several cell cycle-related pathways (Supplementary Fig. 4). This observation is consistent with the widely accepted hypothesis that cell cycle dysregulation is an early event in arsenic-induced carcinogenesis (Al-Eryani et al. 2017; Hunt et al. 2014; States 2015; Tam et al. 2020; Zhou and Xi 2018). Interestingly, the nucleotide excision repair (NER) pathway is induced at 7-wk (Fig. 3B and Supplementary Table 5). As^3+^ exposure is reported to inhibit the function of the NER pathway in human lung fibroblast cell line IMR-90 and mouse primary keratinocytes, albeit with much higher (10–40 μM) acute (24 h) As^3+^ exposure (Holcomb et al. 2017). Furthermore, single nucleotide polymorphisms in several NER pathway genes have been demonstrated to be associated with arsenic-induced skin cancer development (Applebaum et al. 2007; Banerjee et al. 2007) and heightened chromosomal aberration in chronically exposed human populations (Banerjee et al. 2007). It is possible that the induction in the NER pathway (Supplementary Table 5) is a homeostatic response to alleviate the reduced function upon As^3+^ exposure. Notably, in our analysis, the z-score for the NER pathway drops sharply from 6.47 in the differentially expressed mRNA dataset (Supplementary Table 5) to 2.496 in the differentially expressed mRNA targets of differentially expressed miRNA (Supplementary Table 6). This suggests that many of the differentially expressed NER pathway molecules could be modulated by feedback between the protein and mRNA levels independent of miRNA regulation.

Analyses of differentially expressed mRNAs at 19-wk reveal dysregulation of a plethora of transformation-related pathways including Rho-GTPAse signaling and nuclear hormone receptor pathways. Activation of Rho-GTPases is a central and critical event in carcinogenesis (Alan and Lundquist 2013; Aspenstrom 2018). It is therefore not surprising to find a significant inhibition of Rho-GTPase signaling pathway (Fig. 3B and Supplementary Table 5) in our dataset and probably represents a homeostatic mechanism to curb the effects of heightened signaling upon chronic As^3+^ exposure as the cells are undergoing transformation initiation. Furthermore, several nuclear hormone receptor signaling pathways are also predicted to be dysregulated, including androgen signaling, estrogen receptor signaling and prolactin signaling (Fig. 3B, Supplementary Table 5 and Supplementary Fig. 5). These pathways are known to be dysregulated in multiple cancer types including hormone independent melanoma and non-melanoma skin cancers and are often targets for chemotherapeutic intervention (Chan et al. 2018; Dika et al. 2019; Hua et al. 2018; Karayazi Atici et al. 2020; Mal et al. 2020; Pisano et al. 2021; Porter et al. 2019; Rajabi et al. 2017; Xiao et al. 2019). As^3+^ exposure at the environmentally relevant level of 100 nM for 48 h was found to suppress the expression of ERα mRNA and protein in MCF-7 cells and bind competitively to its hormone binding domain with K_i_ of 5 nM, modulating its downstream signaling (Davey et al. 2007; Stoica et al. 2000; Watson and Yager 2007). Furthermore, such low As^3+^ exposure (100–700 nM) using a hepatoma cell line has also been shown to inhibit transcription of androgen receptor signaling modulated target molecules, possibly by interaction with its zinc finger containing DNA binding domain (Bodwell et al. 2006; Watson and Yager 2007). Dysregulation of these pathways are instrumental in EMT (Di Zazzo et al. 2019; Voutsadakis 2016; Yoriki et al. 2019), and perhaps unsurprisingly, we observe first signs of EMT in As^3+^ exposed cells at this time point. Our data thus clearly demonstrate hallmarks of transformation initiation in As^3+^ exposed HaCaT cells at 19-wk.

Data at 28-wk time point also reveal dysregulation of several critical pathways classically associated with a wide variety of cancers. e-NOS signaling, a master regulator of cancer development (Khan et al. 2020) including skin cancer (Bruch-Gerharz et al. 1998; Dhar et al. 2002), is inhibited (Fig. 3B and Supplementary Table 5). Such inhibition is well characterized to promote tumor growth (Xu et al. 2002). Dysregulated NOS signaling in turn leads to ER stress (Gotoh and Mori 2006) and subsequently modulates unfolded protein response (UPR) (Nakato et al. 2015). Both ER stress and UPR are predicted to be dysregulated at 28-wk time point (Fig. 3B, Supplementary Table 5 and Supplementary Fig. 6). ER stress initially brings about induction in the translation of ATF4 and modulation of macroautophagy and apoptosis but this ATF4 induction is abrogated under circumstances of prolonged ER stress (Rozpedek et al. 2016). In agreement, our data show a trend of induction for ATF4 at 7-wk (not significant) with significant suppression at 28-wk (Fig. 4). In addition, we also demonstrate that ER stress is invoked via the PERK arm at 7wk, whereas, at 28-wk, all three arms (PERK, IRE1 and ATF4) are involved. Our data thus reflect prolonged molecular stress that could play a key role in carcinogenesis upon chronic As^3+^ exposure. Dysregulation of ER stress pathway leads to UPR (Pallmann et al. 2019) and cancer (Wortel et al. 2017) both of which are occuring at the 28-wk time point in As^3+^-exposed cells. UPR is inhibited by proteasomal inhibition (Amanso et al. 2011; Lee et al. 2003) and dysregulated proteasome is itself a hallmark of cancer (Morozov and Karpov 2019). UPR thus is a key process bringing about molecular cross-talk between the two arms of protein degradation pathway, viz*.*, ubiquitin-proteasome system and ER stress-induced autophagy. This cross-talk could mean that chronic As^3+^ exposure brings about proteome-wide changes not only by modulating the epigenome and the transcriptome, but also the cellular degradome, as recent studies suggest (Dodson et al. 2018; Tam and Wang 2020).

This interaction may also explain in part why we have some discordance in protein and RNA-seq data for a few of the EMT markers and ER stress molecules we tested (Figs. 1 and 4). Other explanations are also possible. For example, while tarbase predicts that *ERN1* (gene encoding IRE1) is targeted by miR-let7 (Karagkouni et al. 2018), which is induced in our 19 and 28-wk dataset (Supplementary Table 2), IPA does not show this in their miRNA-mRNA expression pairing (Supplementary Table 4). Thus, the predictions of miRNA-mRNA pairing can be discordant in the databases. Additionally, several miRNAs lead to translation repression of the target proteins without altering the mRNA levels (Bhattacharyya et al. 2006; Wilczynska and Bushell 2015), such as those of ZO-1 (mir-34 at 7-wk; miR-let7 at 28-wk), β-Catenin (mir-200a at 28-wk), Slug (miR-218 at 28-wk) and N-Cadherin (mir-181b at 28-wk).

Additionally, this also partly explains why we see a considerable discordance in our miRNA-mRNA expression pairing. Our data demonstrate that several differentially expressed miRNA molecules are potentially able to modulate the expression of one differentially expressed target mRNA molecule (Supplementary Table 4). However, in the cellular context, it is likely that only one of the differentially expressed miRNAs is actually targeting the differentially expressed mRNA molecule rather than all the miRNAs together. For example, in our 7-wk dataset (Supplementary Table 4), AQP3 mRNA is induced and can be targeted by 4 distinct differentially expressed miRNAs (hsa-miR-222, hsa-miR-3661, hsa-miR-663b; all induced and hsa-miR-4688; suppressed). It is possible that in the cells, only the hsa-miR-4688 is actually regulating the expression of AQP3 mRNA, although, all the other three are also potentially capable of doing so. Another possibility is that AQP3 is induced at the mRNA level and the induction of one or more targeting miRNAs (hsa-miR-222, hsa-miR-3661, hsa-miR-663b) is actually a homeostatic response to bring down the expression of AQP3 mRNA to the basal level. Together, these observations suggest that while expression pairing is an important and useful tool to categorize possible miRNA-mRNA interactions in a large dataset, further experimental validation needs to be performed to elucidate the nature of these predicted pairings, both at the mRNA and protein level.

The present work provides a much-needed picture of the sequence of molecular events taking place at each phase of carcinogenic transformation by chronic As^3+^ exposure in a well-established model of arsenic-induced cSCC. The events change dynamically with time and consist of several alterations that are consistent both with As^3+^ exposure as well as established mechanisms of carcinogenesis in the skin and other organs. In addition, we also provide considerable validation of transcriptomic prediction at the protein level in multiple molecules spanning three different branches of the ER stress pathway. This work thus presents a framework for understanding the events leading from exposure initiation through to transformation. Together, our data provide strong evidence as to how different molecules and pathways known to be dysregulated individually in cancer and upon As^3+^ exposure interact as complex coordinated networks (Supplementary Fig. 4–6) to bring about cellular changes at successive steps in the process of As^3+^-induced skin carcinogenesis at toxicologically relevant exposure conditions. This work and the existing dataset open up possibilities of future studies to empirically address the effects of stochasticity in carcinogenesis as well as the efficacy of passage matching for chronic exposure scenarios. Finally, it would be imperative to study proteome-wide changes brought about by chronic As^3+^ exposure in a similar model to understand how the proteomic and transcriptomic changes are correlated in the process of carcinogenesis.

## Supplementary Information

Below is the link to the electronic supplementary material.

Supplementary file1 (DOCX 17 kb)

Supplementary file2 (XLSX 16 kb)

Supplementary file3 (XLSX 32 kb)

Supplementary file4 (XLSX 309 kb)

Supplementary file5 (XLSX 135 kb)

Supplementary file6 (XLSX 20 kb)

Supplementary file7 (XLSX 20 kb)

Supplementary file8 (TIF 45398 kb)

Supplementary file9 (TIF 28141 kb)

Supplementary file10 (TIF 22563 kb)

Supplementary file11 (TIF 64189 kb)

Supplementary file12 (TIF 33342 kb)

Supplementary file13 (TIF 42289 kb)

Supplementary file14 (DOCX 24 kb)

## Data Availability

All the transcriptomic data have been deposited to appropriate databases (accession numbers are provided in the materials and methods sections). All other data/material generated as a part of this work are available upon request.
