# Supplementary figures and images for: Dynamic alteration in miRNA and mRNA expression profiles at different stages of chronic arsenic exposure-induced carcinogenesis in a human cell culture model of skin cancer
Source: Arch Toxicol. 2021 May 25;95(7):2351–65. doi: 10.1007/s00204-021-03084-2 (PMC8241660; doi:10.1007/s00204-021-03084-2)

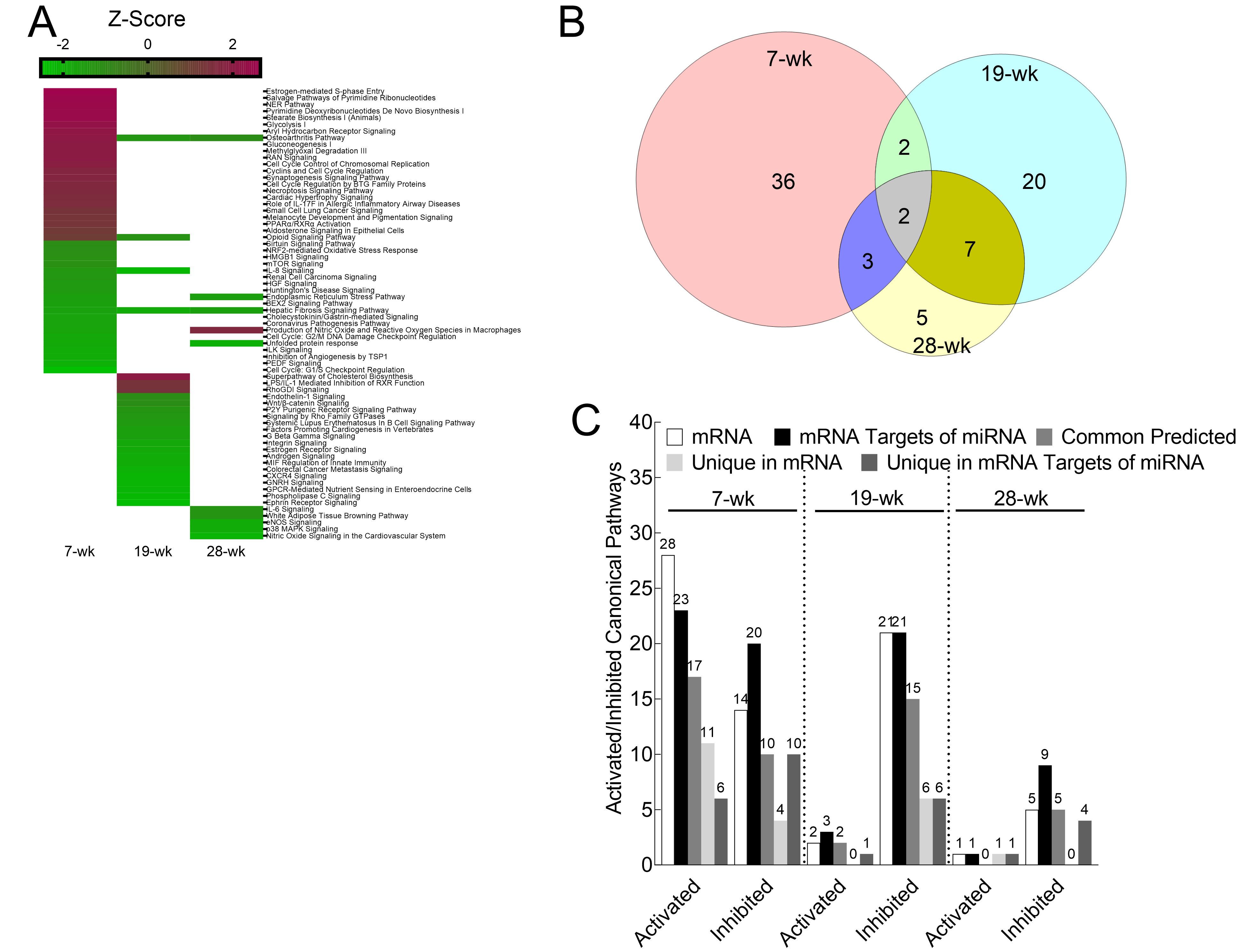

Supplement: Supplementary file 1 — Supplementary file1 (DOCX 17 kb) [file 204_2021_3084_MOESM1_ESM.tif]

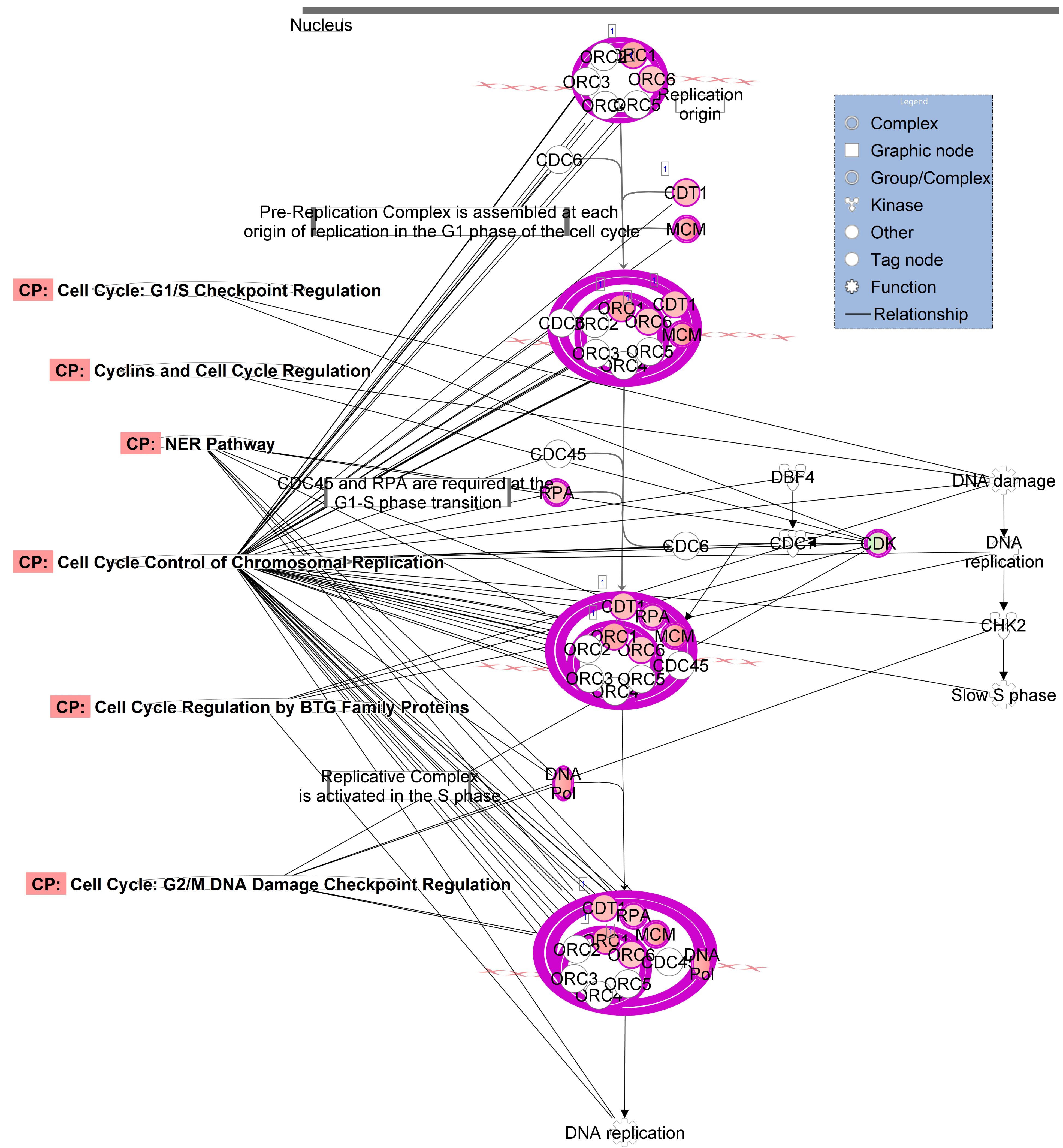

Supplement: Supplementary file 8 — Supplementary file8 (TIF 45398 kb) [file 204_2021_3084_MOESM8_ESM.tif]

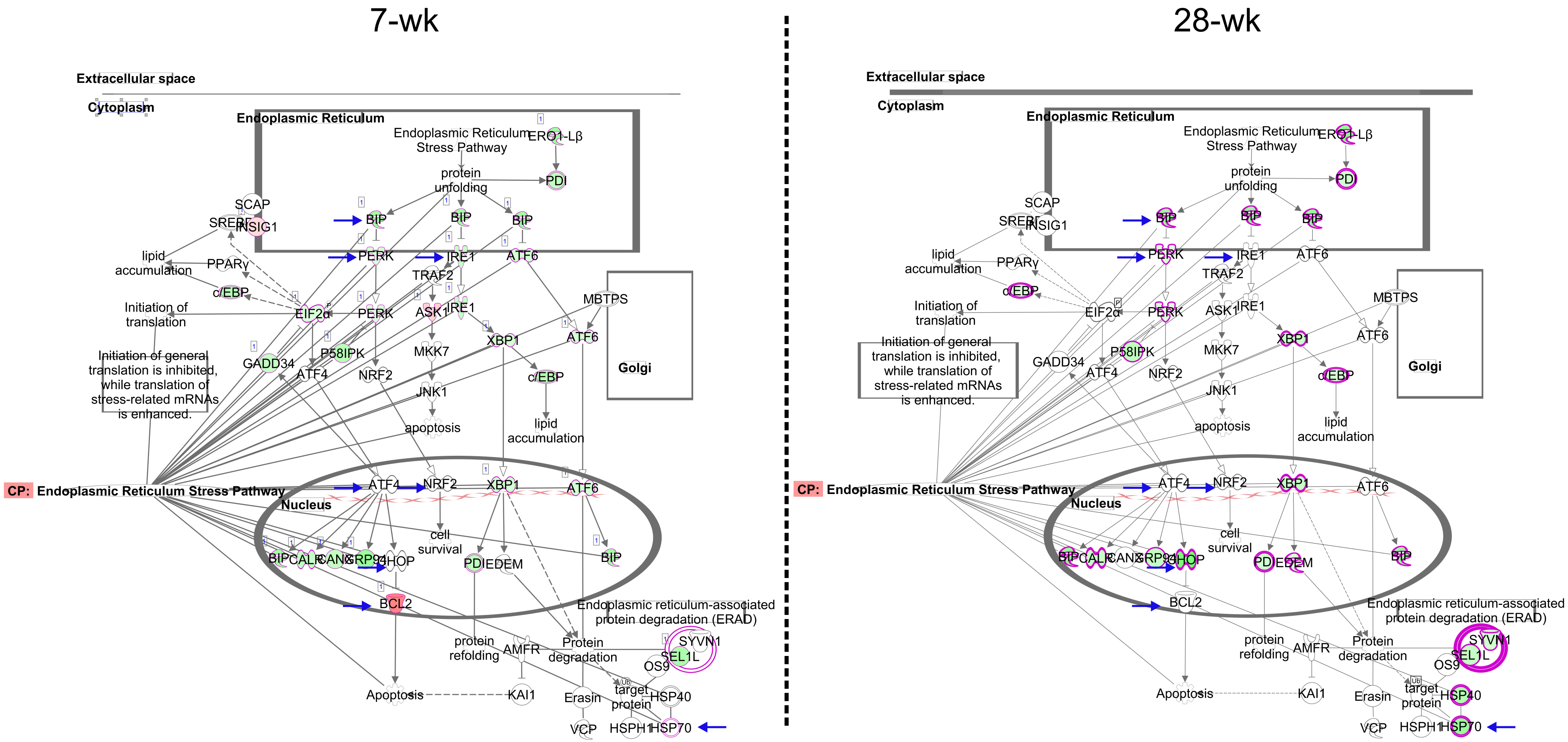

Supplement: Supplementary file 9 — Supplementary file9 (TIF 28141 kb) [file 204_2021_3084_MOESM9_ESM.tif]

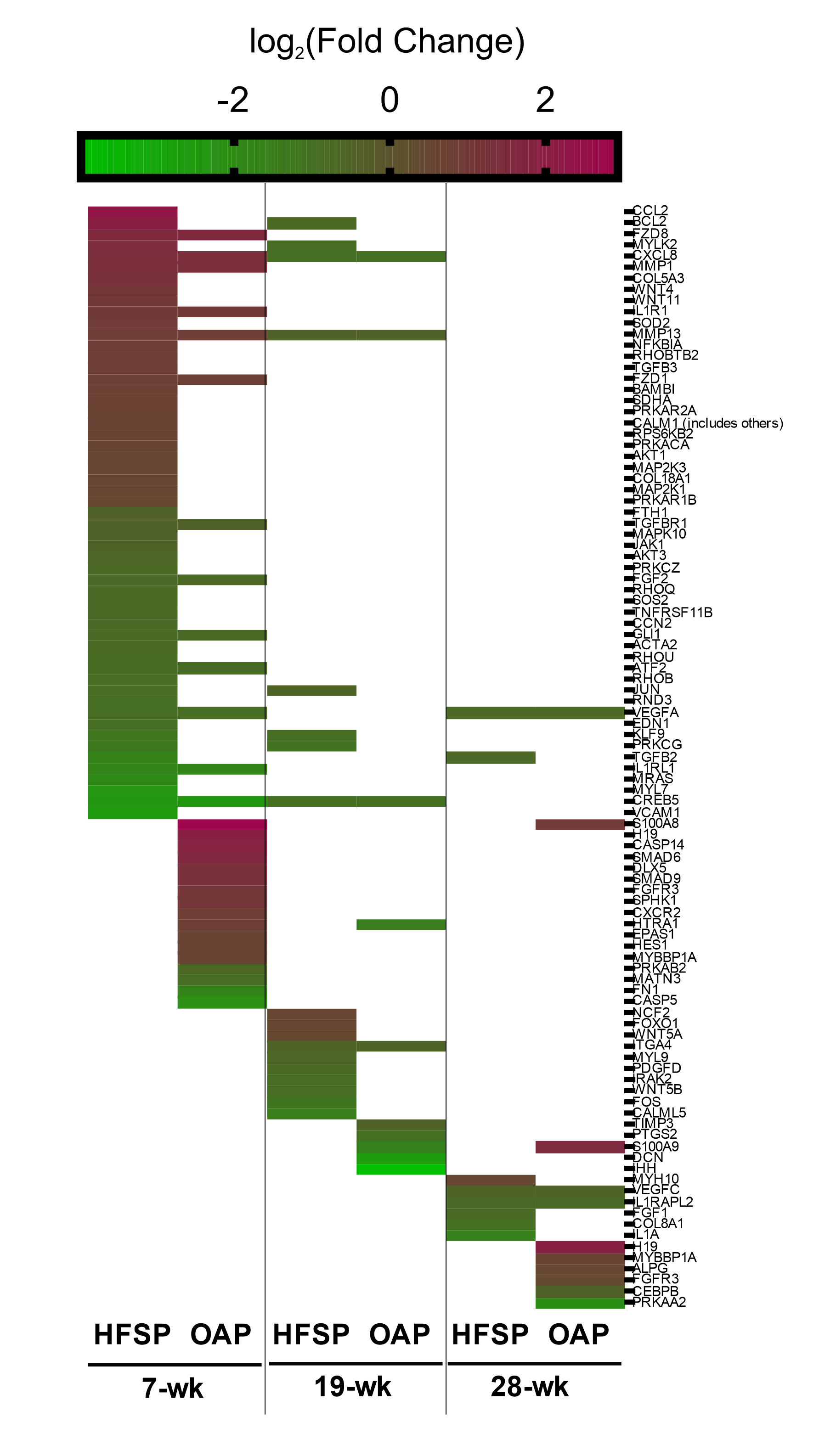

Supplement: Supplementary file 10 — Supplementary file10 (TIF 22563 kb) [file 204_2021_3084_MOESM10_ESM.tif]

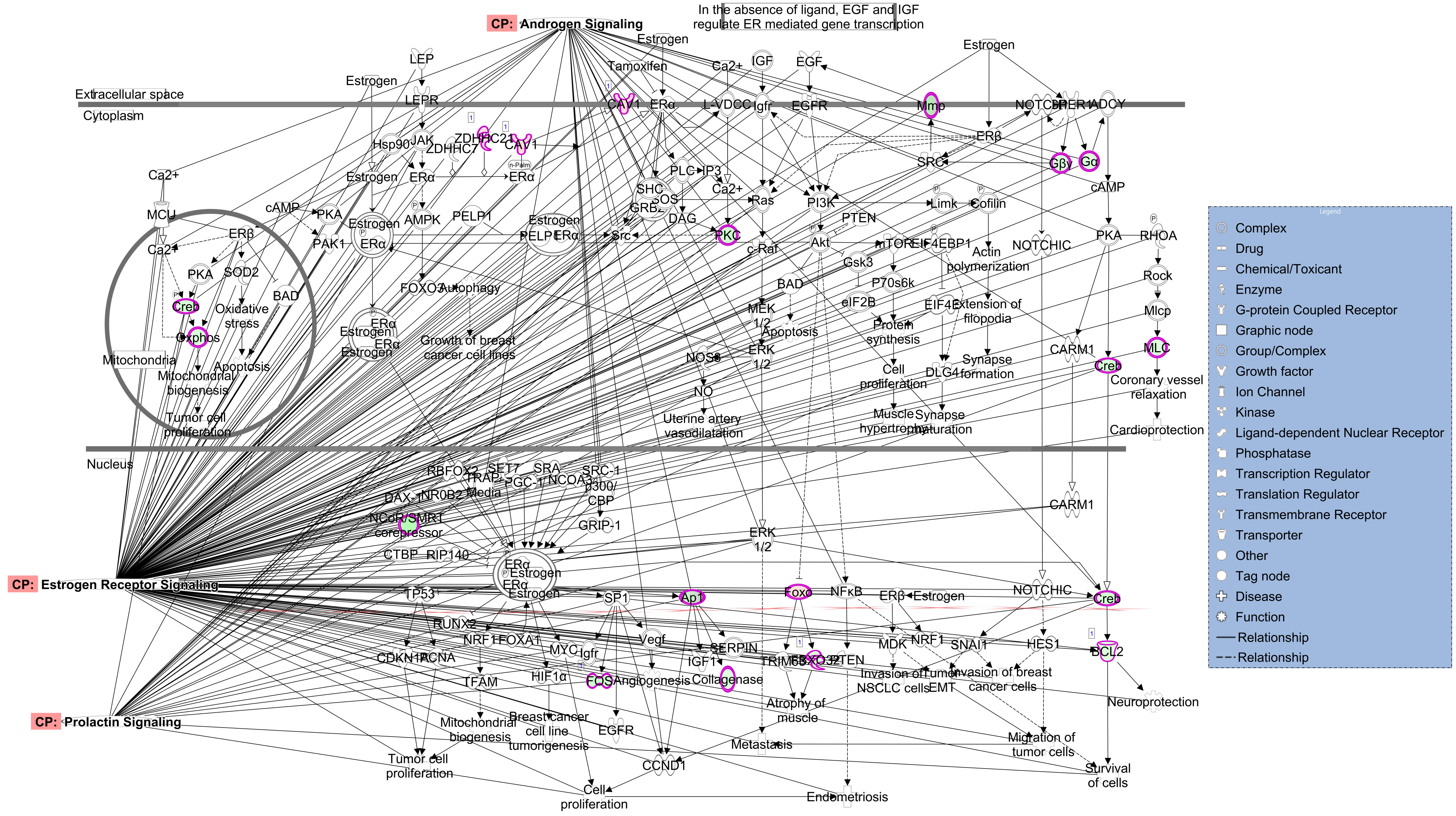

Supplement: Supplementary file 11 — Supplementary file11 (TIF 64189 kb) [file 204_2021_3084_MOESM11_ESM.tif]

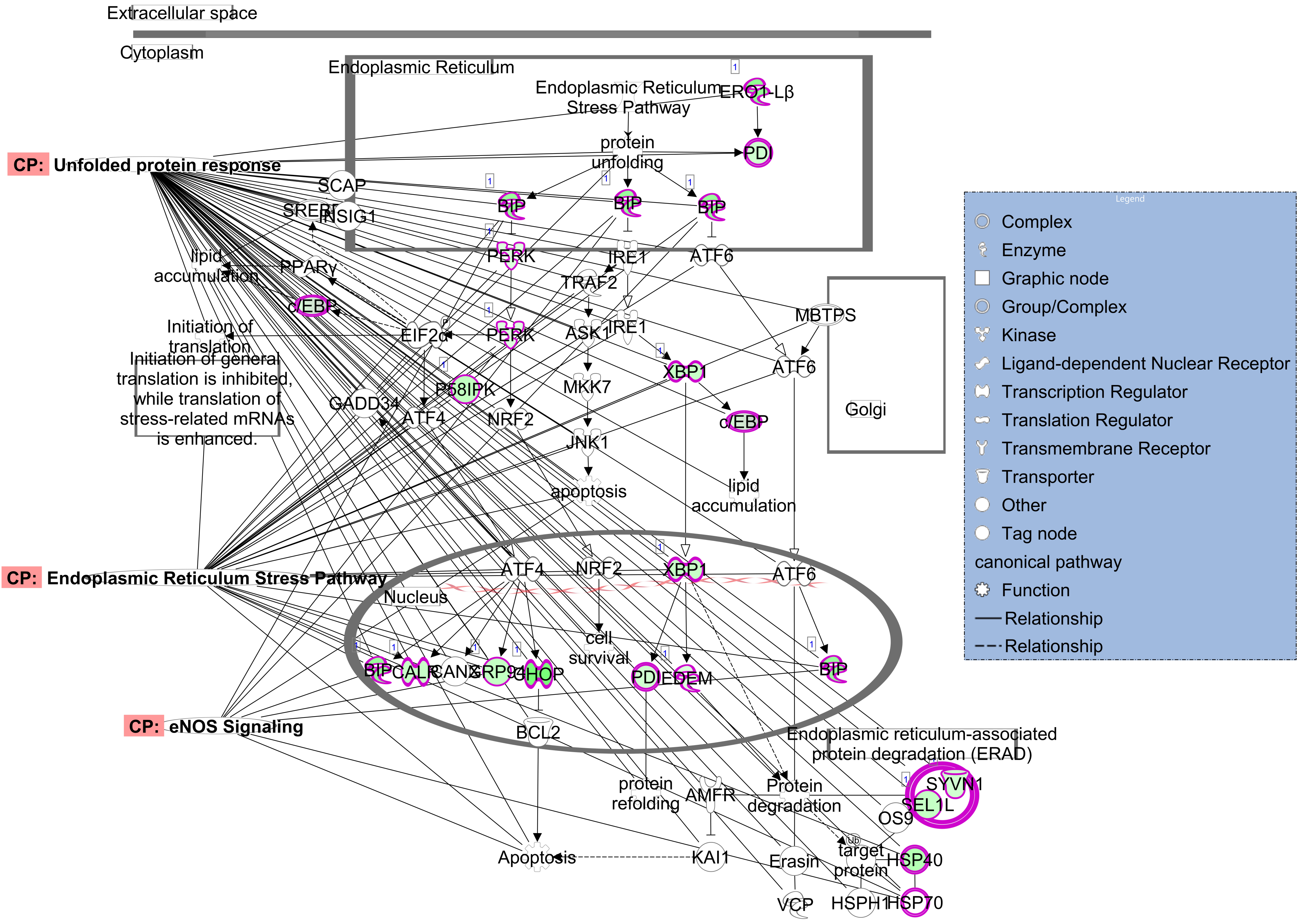

Supplement: Supplementary file 12 — Supplementary file12 (TIF 33342 kb) [file 204_2021_3084_MOESM12_ESM.tif]
