# Supplementary material for: Dynamic alteration in miRNA and mRNA expression profiles at different stages of chronic arsenic exposure-induced carcinogenesis in a human cell culture model of skin cancer
Source: Arch Toxicol. 2021 May 25;95(7):2351–65. doi: 10.1007/s00204-021-03084-2 (PMC8241660; doi:10.1007/s00204-021-03084-2)
Supplement: Supplementary file 13 — Supplementary file13 (TIF 42289 kb) [file 204_2021_3084_MOESM13_ESM.docx]

## miRNA Data Mapping

Paired end RNA-Seq data were generated for each experimental condition. The data for each replicate were stored, trimmed, mapped, and quantified individually. For miRNA, Trimgalore, a wrapper written for the algorithm cutadapt (Martin 2011) was used to trim and quality filter the newly generated reads. The reads were run through the trimming process repeatedly until the output reads were identical to the input reads. This typically took three passes. The miRNA fastq data for each sample then required pre-processing prior to being analyzed using miRDeep2 v:0.0.8 (Friedlander et al. 2012). Specifically, the fastq formatted files were converted to fasta format and the corresponding quality scores were discarded. The fasta definition lines were then truncated at the first whitespace. The reads were then mapped to the NCBI human reference genome version 37.1 (released 2009) using mirDeep2 mapper.pl. An example of the command line:

mapper.pl input.fasta -c -i -j -l 18 -m -p Hs_build-37.1.fa -s \ output.collapsed.fasta -t mappedReads.arf

This created a mapped dataset in .arf format as well as a file of unique miRNAs that appeared in the fasta dataset as well as their count, output.collapsed.fasta, which is used in the next step of the analysis. The program that then identified and quantified the miRNAs was mirDeep2.pl. An example of the command line:

miRDeep2.pl output.collapsed.fasta Hs_build-37.1.fa mappedReads.arf \

mature.Hs.cleaned.fasta mature.non-Hs.cleaned.fasta \

hairpin.Hs.cleaned.fasta -t Human 2>report.log

The mature.Hs.cleaned.fasta, mature.non-Hs.cleaned.fasta, and hairpin.Hs.cleaned.fasta were derived from miRBase as described at https://drmirdeep.github.io/mirdeep2_tutorial.html. The trimmed reads were mapped to the human reference genome hg19 (NCBI build 37.1 released 2009) using the RNA-Seq mapping software TopHat (Trapnell et al. 2012) and annotated transcripts were quantified in units of Fragments Per Kilobase of transcript per Million mapped reads (FPKM) using Cufflinks (Trapnell et al. 2012). The gene annotation used for the quantification was downloaded from ENSEMBL (version 81). This file was filtered to remove all records from the gtf file whose molecular biotype was annotated as either ribosomal RNA (rRNA), or mitochondrial tRNA (Mt_tRNA). The resultant files were used in cufflinks [arguments--GTF Hs_build-37.1.gtf (Mt or rRNA filtered out) --compatible-hits-norm-M Hs_build 37.1_MT_RRNA.gtf (only Mt and rRNA)] and cuffdiff [GTF argument set to Hs_build-37.1.gtf (Mt or rRNA filtered out)] to ensure ribosomal RNA or mitochondrial RNA, that remained after efforts to remove them during the library preparation, would not influence the derived FPKM values. Data have been deposited in the GEO database, accession number GSE153057.

References

Friedlander MR, Mackowiak SD, Li N, Chen W, Rajewsky N (2012) miRDeep2 accurately identifies known and hundreds of novel microRNA genes in seven animal clades. Nucleic Acids Res 40(1):37-52 doi:10.1093/nar/gkr688

Martin M (2011) Cutadapt removes adapter sequences from high-throughput sequencing reads. EMBnetjournal 17:10-12 doi:<https://doi.org/10.14806/ej.17.1.200>.

Trapnell C, Roberts A, Goff L, et al. (2012) Differential gene and transcript expression analysis of RNA-seq experiments with TopHat and Cufflinks. Nat Protoc 7(3):562-78 doi:10.1038/nprot.2012.016
