# Supplementary material for: Dynamic alteration in miRNA and mRNA expression profiles at different stages of chronic arsenic exposure-induced carcinogenesis in a human cell culture model of skin cancer
Source: Arch Toxicol. 2021 May 25;95(7):2351–65. doi: 10.1007/s00204-021-03084-2 (PMC8241660; doi:10.1007/s00204-021-03084-2)
Supplement: Supplementary file 14 — Supplementary file14 (DOCX 24 kb) [file 204_2021_3084_MOESM14_ESM.docx]

# Supplementary Tables and Methods

**Table S1** Details regarding the antibodies used in this study.

**Table S2** Longitudinal representation of differentially expressed miRNAs at 7, 19 and 28-wk (Differentially expressed miRNA is defined as p_Eq<0.05). Row 1 provides information about the table contents. The serial numbers assigned to the differentially expressed miRNAs in this table correspond to the numbers in Fig. 2C.

**Table S3** Longitudinal representation of differentially expressed mRNAs at 7, 19 and 28-wk (Differentially expressed mRNA is defined as p<0.01 and FC>±30%). Row 1 provides information about the table contents. The serial numbers assigned to the differentially expressed miRNAs in this table correspond to the numbers in Fig. 2F.

**Table S4** Expression pairing demonstrating relationships between differentially expressed mRNA targets of differentially expressed miRNAs at 7, 19 and 28 wk. The three sheets in the MS-Excel file correspond with the specific time points analyzed. Row 1 in each sheet provides information about the table contents.

**Table S5** Longitudinal representation of predicted dysregulated pathways based on all differentially expressed mRNA molecules at 7, 19 and 28 wk. Row 1 provides information about the table contents. Activation is defined as -log(p-value)>1.3; Z-score >1, while inhibition is defined as -log(p-value)>1.3; Z-score <-1. The table is arranged in the same order as in Fig. 3B.

**Table S6** Longitudinal representation of predicted dysregulated pathways based on differentially expressed mRNA targets of differentially expressed miRNA molecules at 7, 19 and 28 wk. Row 1 provides information about the table contents. Activation is defined as -log(p-value)>1.3; Z-score >1, while inhibition is defined as -log(p-value)>1.3; Z-score <-1. The table is arranged in the same order as in Supplementary Fig. 1A.**Supplementary Methods**  Details are provided about the data mapping procedure including the bioinformatic pipelines and algorithms used for differential miRNA expression analysis.

# Supplementary Figure Legends

**Fig. S1** Predicted dysregulated pathways based on differentially expressed mRNA targets of differentially expressed miRNA molecules. **a** Heat Map of predicted activated/inhibited pathways at 7, 19 and 28-wk (presented in the same order as in Supplementary Table 6). **b** Venn diagram depicting the distribution of predicted dysregulated pathways [-log(p-value)>1.3; Z-score >±1] at each time point along with the number of overlaps at different time points. **c** Bar graph showing the relationship between the pathways predicted to be activated/inhibited at each time point by differentially expressed mRNA molecules and by differentially expressed mRNA targets of differentially expressed miRNA molecules.

**Fig. S2** Predicted dysregulation of ER stress pathway in As^3+^ exposed HaCaT cells compared to passage matched unexposed controls at 7 and 28-wk. The induced and suppressed mRNA molecules are shown in pink and green fill respectively [intensity of the shade corresponds to the log_2_(Fold Change) of expression]. Any differentially expressed molecule (induced or suppressed) is depicted with single pink outline, while any differentially expressed complex (induced or suppressed) is depicted with double pink outline. Blue arrows indicate the molecules chosen for validation at the protein level by immunoblot in Fig. 4.

**Fig. S3** Heat Map of differentially expressed mRNA molecules belonging to hepatic fibrosis signaling pathway (HFSP) and osteoarthritis pathway (OAP) at 7, 19 and 28-wk. The color code bar on top refers to the log_2_(Fold Change) expression values. Absence of a bar (represented by white) signifies either that mRNA molecule was not detected at that time point or was not differentially expressed at that time point.

**Fig. S4** Predicted dysregulated cell cycle related and DNA repair pathways at 7-wk engage in extensive molecular crosstalk by sharing differentially expressed molecules. Interactions between the following predicted activated/inhibited canonical pathways are shown: cell cycle: G1/S checkpoint regulation, cell cycle regulation by BTG family proteins, cell cycle: control of chromosomal replication, cyclins and cell cycle regulation, cell cycle: G2/M DNA damage checkpoint regulation and NER pathway. The induced and suppressed mRNA molecules are shown in pink and green fill respectively [intensity of the shade corresponds to the log_2_ (Fold Change) of expression]. Presence of both pink and green shades in a complex indicates there are several family members involved, some of which are induced, and some others suppressed (CDK).

**Fig. S5** Predicted dysregulated nuclear hormone receptor pathways at 19-wk engage in extensive molecular crosstalk by sharing differentially expressed molecules. Relationships and interactions between the following predicted activated/inhibited canonical pathways are shown: estrogen receptor signaling, androgen signaling and prolactin signaling. The induced and suppressed mRNA molecules are shown in pink and green fill respectively [intensity of the shade corresponds to the log_2_ (Fold Change) of expression]. Any differentially expressed molecule (induced or suppressed) is depicted with single pink outline, while any differentially expressed complex (induced or suppressed) is depicted with double pink outline.

**Fig. S6** Predicted dysregulated cancer related pathways at 28-wk engage in extensive molecular crosstalk by sharing differentially expressed molecules. Relationship and interactions between the following predicted activated/inhibited canonical pathways are shown: unfolded protein response, protein ubiquitination pathway and e-NOS signaling. The suppressed mRNA molecules are shown in green fill [intensity of the shade corresponds to the log_2_ (Fold Change) of expression]. Please note that none of the molecules in these pathways are not being induced. Any differentially expressed molecule (induced or suppressed) is depicted with single pink outline, while any differentially expressed complex (induced or suppressed) is depicted with double pink outline.
